# Structural and Functional Characterization of the Protein Kinase Mps1 in *Arabidopsis thaliana*

**DOI:** 10.1371/journal.pone.0045707

**Published:** 2012-09-26

**Authors:** Eduardo Alves Gamosa de Oliveira, Nelilma Correia Romeiro, Elane da Silva Ribeiro, Claudete Santa-Catarina, Antônia Elenir Amâncio Oliveira, Vanildo Silveira, Gonçalo Apolinário de Souza Filho, Thiago Motta Venancio, Marco Antônio Lopes Cruz

**Affiliations:** 1 Laboratório de Biotecnologia Vegetal, Núcleo em Ecologia e Desenvolvimento Sócio-ambiental de Macaé, Universidade Federal do Rio de Janeiro, Macaé, Rio de Janeiro, Brazil; 2 Laboratório Integrado de Computação Científica, Núcleo em Ecologia e Desenvolvimento Sócio-ambiental de Macaé, Universidade Federal do Rio de Janeiro, Macaé, Rio de Janeiro, Brazil; 3 Laboratório de Química e Função de Proteínas e Peptídeos, Centro de Biociências e Biotecnologia, Universidade Estadual do Norte Fluminense Darcy Ribeiro, Campos dos Goytacazes, Rio de Janeiro, Brazil; 4 Laboratório de Biotecnologia, Centro de Biociências e Biotecnologia, Universidade Estadual do Norte Fluminense Darcy Ribeiro, Campos dos Goytacazes, Rio de Janeiro, Brazil; 5 Laboratório de Biologia Celular e Tecidual, Centro de Biociências e Biotecnologia, Universidade Estadual do Norte Fluminense Darcy Ribeiro, Campos dos Goytacazes, Rio de Janeiro, Brazil; Texas A&M University, United States of America

## Abstract

In eukaryotes, protein kinases catalyze the transfer of a gamma-phosphate from ATP (or GTP) to specific amino acids in protein targets. In plants, protein kinases have been shown to participate in signaling cascades driving responses to environmental stimuli and developmental processes. Plant meristems are undifferentiated tissues that provide the major source of cells that will form organs throughout development. However, non-dividing specialized cells can also dedifferentiate and re-initiate cell division if exposed to appropriate conditions. Mps1 (**M**ono**p**olar **s**pindle) is a dual-specificity protein kinase that plays a critical role in monitoring the accuracy of chromosome segregation in the mitotic checkpoint mechanism. Although Mps1 functions have been clearly demonstrated in animals and fungi, its role in plants is so far unclear. Here, using structural and biochemical analyses here we show that Mps1 has highly similar homologs in many plant genomes across distinct lineages (e.g. AtMps1 in *Arabidopsis thaliana*). Several structural features (i.e. catalytic site, DFG motif and threonine triad) are clearly conserved in plant Mps1 kinases. Structural and sequence analysis also suggest that AtMps1 interact with other cell cycle proteins, such as Mad2 and MAPK1. By using a very specific Mps1 inhibitor (SP600125) we show that compromised AtMps1 activity hampers the development of *A. thaliana* seedlings in a dose-dependent manner, especially in secondary roots. Moreover, concomitant administration of the auxin IAA neutralizes the AtMps1 inhibition phenotype, allowing secondary root development. These observations let us to hypothesize that AtMps1 might be a downstream regulator of IAA signaling in the formation of secondary roots. Our results indicate that Mps1 might be a universal component of the Spindle Assembly Checkpoint machinery across very distant lineages of eukaryotes.

## Introduction

Protein phosphorylation and dephosphorylation are among the most prominent and widespread post-translational modifications, being an essential part of most regulatory signaling cascades in eukaryotes and prokaryotes [1]. Eukaryotic protein kinases (EPKs) catalyze the transfer of a γ-phosphate from ATP (or GTP) to a specific amino acid in the protein substrate (typically serine, threonine and/or tyrosine) [1], [2]. EPKs have evolved from simpler eukaryotic-like kinases that are widespread, although not well-characterized, in prokaryotes [2], [3]. Some lines of evidence suggest a positive correlation between the number of protein kinases and complexity (i.e. multicellularity) in prokaryotes [4], [5]. Although in eukaryotes there is no such correlation, eukaryotic genomes typically harbor highly expanded protein kinase repertoires when compared to their prokaryotic counterparts [4], [6].

In plants, EPKs have been implicated in signaling cascades that mediate responses to environmental stimuli and developmental processes [7]–[10]. Many of these signaling pathways can directly affect cell cycle regulation [11]–[13], such as the MAPK pathway – a major regulator of development, immunity and stress responses in plants [14]. Plant and animal cell cycle biochemistry share several common regulators, such as the cyclins, cyclin-dependent kinases (CDKs) and CDK inhibitors [15]–[20]. It has been recently shown that the premature interaction between NACK1 and NPK1 (MAPKKK) is prevented by CDK-mediated phosphorylation, a critical step for regulating the timing of cytokinesis [21]. Several retinoblastoma-related proteins are also phosphorylated by cyclin-CDK complexes during specific cell cycle stages [22]. Dozens of yeast CDK targets have been identified and most of them participate in the cell cycle progress (e.g. DNA replication and chromosome segregation) [23]. Although the complexity of the cyclin family is arguably higher in plants than in animals, plant CDK targets remain elusive [24]. Conversely to the many similarities discussed above, there are also remarkable differences between the animal and plant cell cycle, especially with regard to the metaphase plate formation and microtubule arrangement during cytokinesis [25].

Mps1 (**M**ono**p**olar **s**pindle) kinase family members are characterized by a C-terminal, dual-specificity protein kinase domains [26]. They typically have divergent N-terminal regions, lacking clear unifying motifs [26]. Mps1 was initially characterized as a critical player in centrosome (spindle-pole) duplication and proper formation of the spindle pole body [27]–[29]. Curiously, this role has proven controversial and not unambiguously demonstrated outside budding yeast [30], [31]. Other studies consistently demonstrated, across several eukaryotes, that Mps1 participate in the mitotic checkpoint, which monitors the accuracy of chromosome segregation [32]–[36]. Structural and functional studies showed that the human Mps1 (hMps1) is phosphorylated at multiple amino acids by several distinct kinases, such as Cdk1, MAPK, Plk1 and hMps1 itself, revealing a complex regulatory landscape [37]–[39]. Due to this prominent role in controlling the cell cycle, Mps1 has been considered a potential target for antineoplastic drugs and novel Mps1 inhibitors have been tested over the past decade [40]–[45].

Plants typically keep sets of undifferentiated cells (i.e. meristems), which are the most important source of cells that will constitute organs throughout development. However, it has been long demonstrated that non-dividing specialized cells can dedifferentiate and re-initiate cell division; not only in normal developmental stages (e.g. lateral root formation), but also during regeneration from injury or exposure to growth regulators [20], [46]. The shift from differentiated to dedifferentiated phenotypes is very complex and not totally understood, although some works support pervasive chromatin modification and drastic change in the transcriptional landscape preceding the cell cycle entry [46], [47].

While the primary root develops during embryogenesis, the secondary roots start from asymmetric divisions of the cells in the pericycle [48], [49], in a process that is induced by the auxin IAA [46], [50], [51]. Although some important studies shed light on several aspects of lateral root formation [50]–[53], the molecular steps to reactivate the cell cycle at the pericycle is not totally understood.

In the present work we use biochemical, structural and computational analyses to show that the protein kinase encoded by the gene At1G77720 is a plant ortholog of Mps1 with critical roles in the cell cycle regulation, suggesting the universality of Mps1 as a critical cell cycle checkpoint protein.

**Figure 1:**
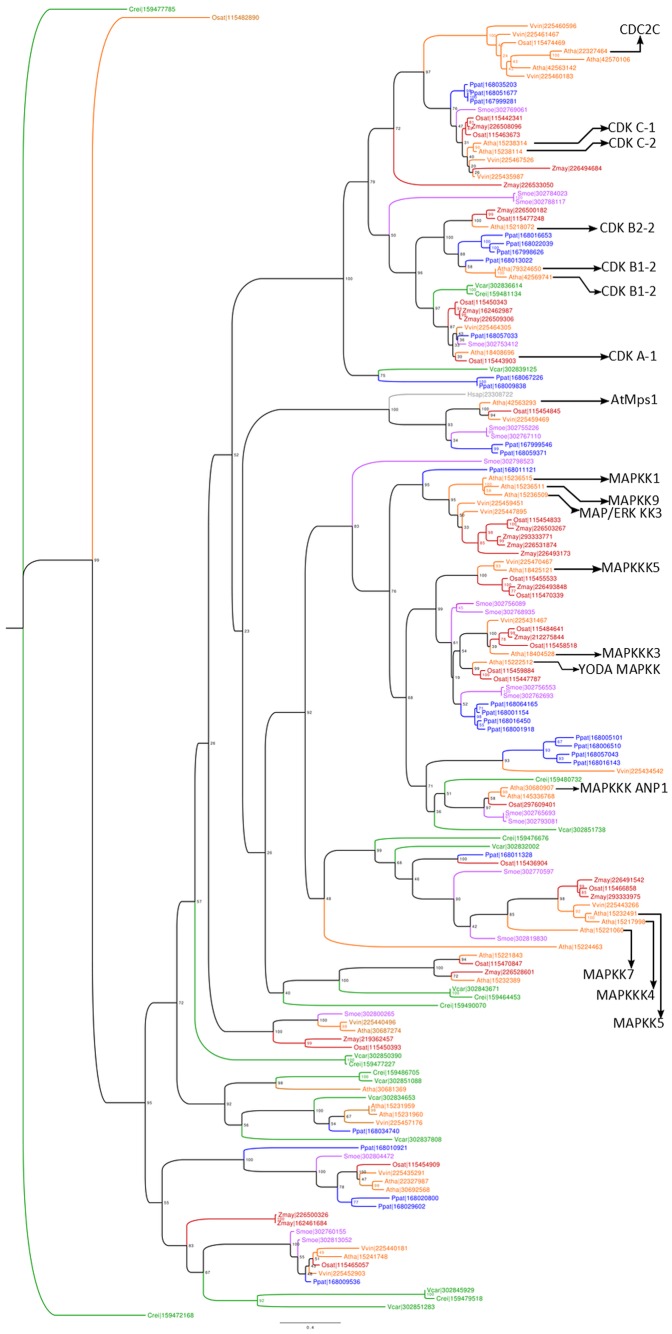
Phylogenetic reconstruction of the plant Mps1 homologs. The tree was computed using the maximum-likelihood method. Internal nodes were labeled with bootstrap support values. Colors: red (monocots), orange (eudicots), blue (moss), green (green algae), purple (basal vascular plant). Proteins are identified by GI numbers following abbreviated species names. Species list: *Arabidopsis thaliana* (Atha), *Vitis vinifera* (Vvin), *Zea mays* (Zmay), *Oryza sativa* (Osat), *Chlamydomonas reinhardtii* (Crei), *Physcomitrella patens* (Ppat), *Selaginella moellendorffii* (Smoe), *Volvox carteri* (Vcar).

## Results and Discussion

### Conservation and Evolution of Mps1 Homologs in Plants

We searched the *A. thaliana* genome for Mps1 homologs using the amino acid sequence of *Homo sapiens* Mps1 (hMps1; gi:23271249) as BLAST query (see methods for details). The protein kinase AT1G77720 was found as the best hit and used for screening a selected group of genomes including green algae, basal (non-vascular) plants, monocots and eudicots (see methods for a complete list of species). We retained close plant Mps1 homologs by using query and hit (subject) 30% coverage thresholds. To ensure higher quality and reduced redundancy, only sequences from the RefSeq database were used for phylogenetic analysis. We found 156 plant Mps1 homologs that, along with the hMps1, were submitted to multiple sequence alignment and the conserved region (which includes the kinase domain) used for phylogenetic reconstructions.

**Figure 2:**
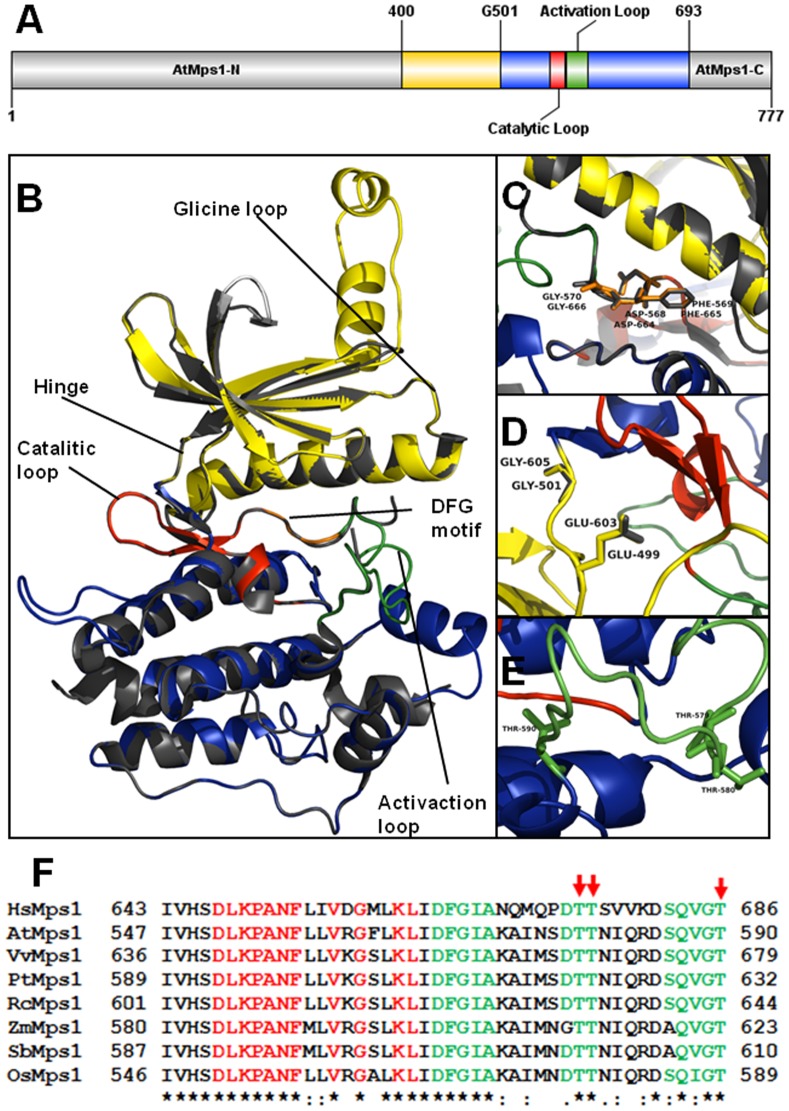
Major structural features of AtMps1. **A)** Linear representation of AtMps1 domains along the full length sequence (777 amino acid residues) (gray). The kinase domain is composed of a small lobe (yellow) and a big lobe (blue) separated by a glycine (G501); **B)** Tridimensional modeling and structural comparison of AtMps1 (gi 28416703) and hMps1 (in gray) (gi 23271249). Protein structures were retrieved from PDB; **C)** Detailed representation of the DFG motif; **D)** Hinge between the N- and C-terminal regions. The critical amino acid residues are E499/G501 in AtMps1 and E603/G605 in hMps1 (shown in gray); **E)** Threonine triad probably involved in autophosphorylation; **F)** Multiple sequence alignment of the catalytic (red) and activation (green) sites from several monocots, eudicots and hMps1. Red arrows mark conserved threonine residues in the catalytic site. Protein GI numbers: hMps1 (*Homo sapiens*; 23271249); AtMps1 (*A. thaliana*; 28416703); PtMps1 (*Populus trichocarpa*; 224063138); RcMps1 (*Ricinus communis*; 255545510); OsMps1 (*Oryza sativa* Indica Group; 125545426); SbMps1 (*Sorghum bicolor*; 242038411).

The reconstructed phylogenetic tree comprises proteins from major protein kinase kinase clades (e.g. CDKs and MAPKs) (Figure 1). The clade containing hMps1 has only one *A. thaliana* gene product, AT1G77720, supporting the result obtained from pairwise comparisons (i.e. BLAST), which showed this gene as the *A. thaliana* best blast hit of hMps1. In this work, AT1G77720 is henceforth called AtMps1. Interestingly, this clade is free of recent duplications in the flowering plants (i.e. angiosperms), which might be a result of conserved functionality and susceptibility to increased gene dosage coming from duplication events. Conversely, there are a series of independent duplication events, including duplications predating the origin of angiosperms and specific duplications after the split of monocots and eudicots (Figure 1). Remarkably, there are several independent lineage-specific duplications of Mps1 members in the moss genome, suggesting an increased functional diversification of the family in non-vascular plants (i.e. mosses) after the split of the vascular plant lineage.

**Figure 3:**
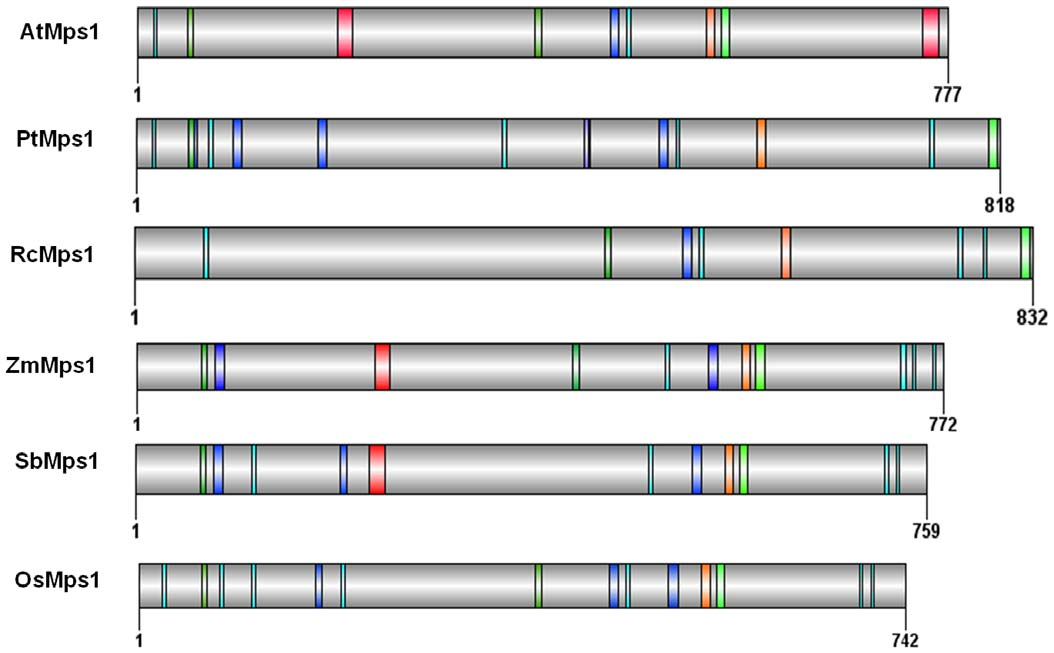
Eukaryotic linear motifs of several Mps1 plant orthologs. Color codes: LIG_APCC_Dbox_1 (light green); Cyclin recognition site LIG_CYCLIN_1 (cyan); MAD2 binding motif LIG_MAD2 (orange); MAPK docking motif LIG_MAPK_1 (blue), NES Nuclear Export Signal TRG_NES_CRM1_1 (red), NLS classical Nuclear Localization Signals (green). AtMps1 (*A. thaliana*; 28416703); PtMps1 (*P. trichocarpa*; 224063138); RcMps1 (*R. communis*; 255545510); OsMps1 (*O. sativa* Indica Group; 125545426); SbMps1 (*S. bicolor*; 242038411).

### Structural Analysis of AtMps1

AtMps1 is encoded by the gene At1G77720, which is located at chromosome 1 and harbors 6 exons. Its protein product has a total of 777 amino acids with a canonical N-terminal protein kinase domain composed of 293 amino acids [1]. The kinase domain has 5 five antiparallel N-terminal β-sheets and 6 C-terminal α-helices. This structure is highly similar to the experimentally derived hMps1 kinase structure (Figure 2) [54]. AtMps1 has several major protein kinase features such as the DFG motif (D568, F569, G570), which is important for the catalytic loop structural conformation (Figure 2). The glycine of the DFG motif confers flexibility to hMps1, a critical requirement for the formation of the catalytic loop [54]. D664 (D568 in AtMps1) coordinates a magnesium ion required for ATP hydrolysis [55]. In the loop between the N- and C-terminal lobes, E499 and G501 form a hinge that is structurally similar to that formed by E603 and G605 in hMps1, implying that the articulation structure of the N- and C-terminal lobes is also conserved between hMps1 and AtMps1 (Figure 2). Three threonine residues are critical for autophosphorylation at hMps1 activation loop: T675, T676 and T686. All these three residues conserved in AtMps1 (T579, T580 and T590) (Figure 2), suggesting that that autophosphorylation is also conserved and important for the regulation of AtMps1.

**Figure 4:**
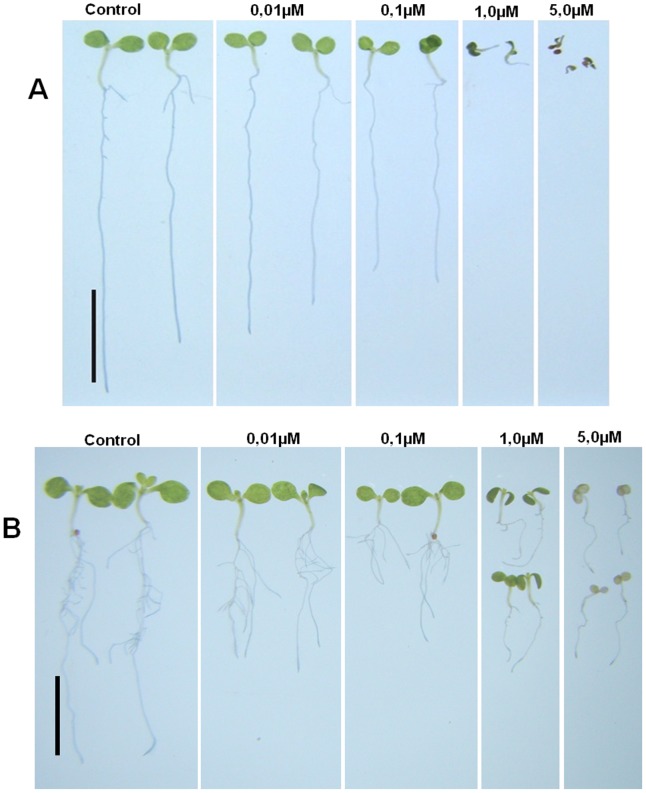
Effect of AtMps1 inhibition on 7-day *A. thaliana* seedlings germinated in MS medium with the Mps1 inhibitor SP600125. **A)** Seedlings without exogenous IAA; **B)** Seedlings incubated for 24 hours with 5 µM of IAA. In both panels, A and B, control seedlings were not exposed to SP600125 and their growth was compared with increasing concentrations of SP600125Black bar: 0,5 cm.

**Figure 5:**
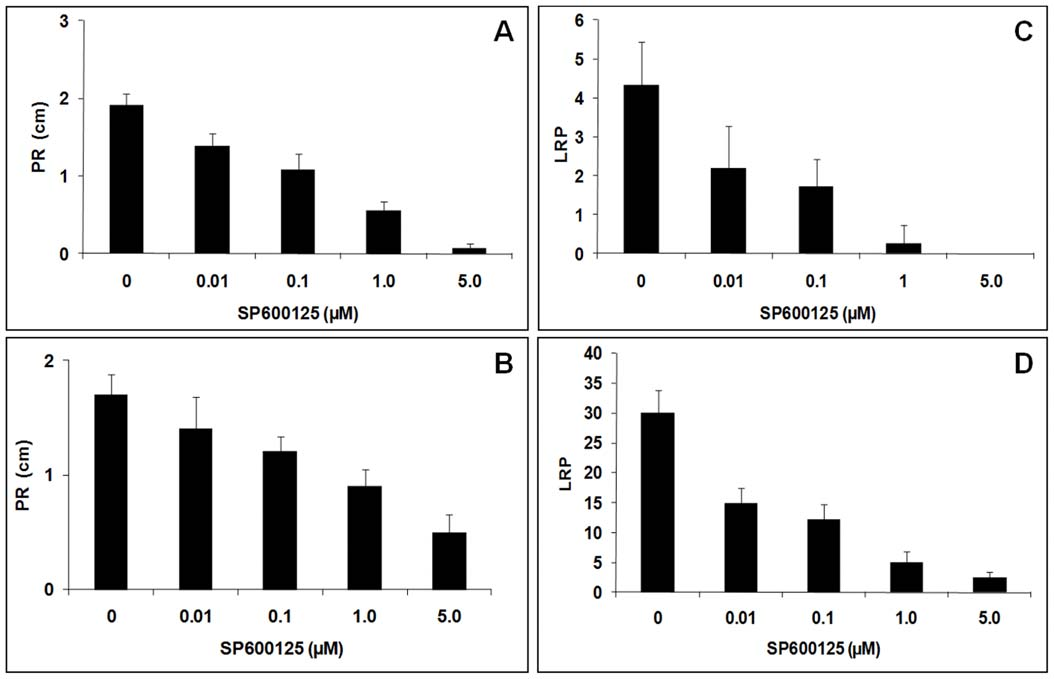
Effect of different concentrations of the Mps1 inhibitor SP600125 on *A. thaliana* root growth. **A)** Primary root length with no IAA preincubation; **B)** Number of visible lateral root primordia with no IAA preincubation; **C)** Primary root length after 24-hour preincubation with 5 µM IAA; **D)** Number of visible lateral root primordia after 24-hour preincubation with 5 µM IAA.

Phosphorylation of several specific residues has been shown to play a major role in Mps1 regulation in yeast and *Xenopus* [56], [57]. In humans, hMps1 phosphorylation is intimately correlated with APC/C mediated ubiquitination and proteasomal degradation [58]. We used three different methods to predict the phosphorylation sites of plant Mps1 homologs [59]–[61]. We found 141 predicted phosphorylation sites in AtMps1, from which 50 are conserved in hMps1 (Table S1). Moreover, several of these residues had their phosphorylation status experimentally demonstrated in hMps1 (e.g. S321 in hMps1; S289 in AtMps1) [37], [62]. Notably, the N-terminal domain of Mps1 has a high density of phosphorylation sites, suggesting an increased regulatory potential (Table S1).

In hMps1, the amino acid T676 (T580 in AtMps1) is a phosphorylation site with regulatory implications concerning the recruitment of Bubr1 to the kinetochore [34], [38], [63], [64]. The interaction and co-localization of Bubr1 and Mps1 at the kinetochore has been demonstrated in several eukaryotes [34], [65]–[67] and our structural analyses indicate that this interaction is preserved in the plant lineage. The residue T686 (T590 in AtMps1) is important for structural integrity, autophosphorylation and transphosphorylation of hMps1 [38], [39], [54]. AtMps1 has been recently shown to autophosphorylate *in vitro* [68], implying that the auto-regulatory step is also retained in plants. Overall, the conservation in *A. thaliana* of all the major structural features responsible for Mps1 functions in yeast and human strongly suggest that its prominent biochemical functions are preserved in the plant lineage and might have been present in early eukaryotic organisms.

### Linear Amino Acid Motifs Provide Important Clues on AtMps1 Functions

Linear motifs are short amino acid modules that are frequently part of regulatory proteins, providing interaction interfaces in protein structures. We searched for such motifs in several plant Mps1 orthologs using the ELM database [69] (Figure 3). We found motifs that could potentially mediate interactions with cyclins, MAD2, APC/C and MAPK – cell cycle regulators that are present in all the investigated plants. It has been recently shown that hMps1 is important for Mad2 recruitment to the kinetochore [43]. Mad2 is a critical component of the Spindle Assembly Checkpoint (SAC), which prevents anaphase onset until all chromosomes are properly attached to the spindle [70]. Mad1 and Mad2 homologs have been characterized in plants and were shown to interact with the nuclear pore complex [71]. The motif LIG_MAPK_1 was also found in all the analyzed plant Mps1 orthologs. Previous studies showed that Mps1 is phosphorylated by MAPK and this modification could be responsible for the interaction between Mps1 and the kinetochore [57]. In addition, AtMps1 harbors a LIG_CYCLIN_1 motif, suggesting that it might be also phosphorylated by cdc28/CDK1, as previously demonstrated in yeast [56]. The presence of the LIG_APCC_Dbox_1 motif in AtMps1 indicates that it could be among the many cell cycle proteins ubiquitinated by the APC/C complex and degraded by the proteasome system [20], [72].

In addition to the protein interaction motifs, AtMps1 have sub-cellular localization modules (i.e. TRG_NES_CRM1_1, TRG_NLS_MonoExtC_3 and TRG_NLS_MonoExtN_4) that corroborate the nucelocytoplasmic functions discussed above. Interestingly, the TRG_NLS_MonoExtN_4 motif can be reversibly inactivated, allowing the protein to operate in the cytoplasm. This domain has been associated with subcellular localization of CDKs in plants [73]. We hypothesize that the presence of such motifs in AtMps1 is directly related to its translocation between cytoplasmic and nucleus, allowing the phosphorylation of specific targets in either cellular component at specific cell cycle phases.

### AtMps1 Activity is Critical for the Development of *A. thaliana* Seedlings

AtMps1 is highly transcribed in 7-day *A. thaliana* seedlings, notably in apical shoot and root meristems, where the cell cycle is highly active to generate new plant aerial and underground tissues (Figure S1) [74], [75]. Conversely, AtMps1 transcription clearly decreases in most differentiated tissues. Moreover, AtMps1 is highly transcribed in the pericycle – a parenchymal layer of cells responsible for lateral root development [76]. Using *A. thaliana* cell suspension cultures Menges et al. showed that AtMps1 (At1G77720) is transcribed at the G_2_ phase [77]. G2 transcription of Mps1 was also demonstrated in other eukaryotes [36], [66], [78], [79], underscoring the importance of Mps1 in the G_2_/M transition in distantly related species. Using genome-wide datasets we were able to find that the transcriptional levels of Mps1 homologs in other plants (eudicots and monocots) (i.e. *Glycine max, Populus trichocarpa*, *Medicago truncatula*, *Oryza sativa*) are also over-expressed in many instances where there is intense cell cycle activity [80], [81]. Prediction of sub-cellular localization and nuclear export motifs indicate that AtMps1 operates mainly in the nucleus, but can also localize to the cytoplasm, as observed in human HeLa cells [32].

The use of small bioactive molecules to study the cell cycle in plants has been proposed as a powerful method to untangle the functions of different signaling components [82]–[84]. It has been previously shown that the inhibitor SP600125 specifically inhibit hMps1 in a dose-dependent fashion [55]. Considering the high sequence and structural similarity between AtMps1 and hMps1, we tested the effects of SP600125 in *A. thaliana* seedlings, especially on the root system architecture [85]. SP600125 hampers the primary and secondary root growth, implying that AtMps1 activity is important for proper development of these tissues (Figure 4 and 5). Moreover, the effect of SP600125 is clearly stronger in secondary than in primary root growth (Figure 4 and 5). This might be due to either a higher accessibility of pericycle cells to the inhibitor or to a higher transcription of AtMps1 in the internal tissue layers, requiring more SP600125 to neutralize AtMps1 activity. Secondary roots originate from pericycle cells arrested at G2 [48], the same cell cycle phase in which AtMps1 is preferentially expressed [77]. Hence, our results indicate that SP600125 blocks the G2-M transition by specifically inhibiting AtMps1 activity and compromising the G2-M transition.

Phytohormones regulate and integrate various signaling cascades involved in endogenous (e.g. development) and environmental processes (e.g. predation, water stress) [86], [87]. Auxins, gibberellins and brassinosteroids control cellular elongation and proliferation [88]. The auxin IAA has been classically shown to activate the formation of secondary roots [48], [50], [51]. Here we show that IAA administration can reverse the AtMps1 inhibition phenotype (Figure 4 and 5), suggesting that this gene might be a cell-cycle regulator acting downstream to the IAA-signaling pathway. Interestingly, IAA regulation of lateral root formation is particularly important when young leaf primordia form and are able to synthesize the hormone, enabling the balance between carbon and nitrogen metabolism through a coordinated development of leaves and roots. The high transcription levels of AtMps1 in the hypocotyl, shoot apical and root meristems further support the roles of AtMps1 as a downstream effector of auxin signaling in critical developmental processes requiring precise cell cycle regulation, probably as a checkpoint protein that prevents anaphase onset with incorrect chromosomal attachment to the spindle [70].

### Conclusion and Future Perspectives

In the present work we analyzed the structure and function of the *A. thaliana* Mps1 ortholog, AtMps1. The high conservation in plants of all major structural features described in other eukaryotes, namely the C-terminal kinase domain, the DFG domain and the threonine triad, responsible for activation by autophosphorylation. Taken together, these observations imply that Mps1 functions are deeply preserved in divergent eukaryotes. Therefore, Mps1 is a cell cycle regulator with major roles in important developmental processes, as observed in other eukaryotic lineages, probably operating as a universal component of the “Spindle Assembly Checkpoint” machinery. Although AtMps1 phosphorylation targets remain to be identified, we hypothesize it is a downstream player of auxin signaling, working as a critical cell cycle regulator during aerial and underground tissue development.

## Materials and Methods

### Databases and Sequence Analysis

Mps1 homologs were detected using BLAST [89], with a minimum coverage threshold of 30% (query and hit). The genomes used in our work were the following: *Chlamydomonas reinhardtii* and *Volvox carteri* (green algae), *Physcomitrella patens* (moss; Bryophyta), *Selaginella moellendorffii* (ancient vascular plant; lycophyte), *Zea mays* and *Oryza sativa* (monocots) and *A. thaliana* and *Vitis vinifera* (eudicots). Multiple sequence alignments were computed using MUSCLE and visualized using Jalview [90]. Phylogenetic reconstructions were performed using RAxML [91].

Linear protein interaction motifs were detected using the Eukaryotic Linear Motif Database (http://elm.eu.org/) [69]. Phosphorylation sites were predicted using were predicted using three distinct methods: PlantPhos, a tool developed to predict phosphorylation sites in plant proteins [61]; MUSITE, which also have some parameters that can be adjusted to analyze plant proteins [59]; and DISPHOS, a method that explicitly uses “intrinsically disordered regions” information to aid the prediction of phosphorylation sites [60]. The non-redundant union of the results obtained using these three independent methods were considered our set of predicted phosphorylation sites. In addition, two independent methods were used to predict kinase families potentially regulating AtMps1 [92], [93]. Subcellular localization was predicted using MultiLoc [94], PSORT [95] and CELLO [96]. Gene expression data for AtMps1 were obtained from the Electronic Fluorescence Pictograph Browser [97] (http://bar.utoronto.ca/efp/cgi-bin/efpWeb.cgi).

### Structural Analysis

The 3D homology model of AtMps was constructed using the amino acid sequence obtained from public databases (AT1G77720). Sequence similarity searches were conducted using BLAST [89]. Based on BLAST scores and structure resolution (2.88 Å), the crystal structure of human Mps1 catalytic domain (T686A mutant) in complex with SP600125 inhibitor (PDB accession 2ZMD) [55], [98] was chosen as template. The 3D homology model was constructed with the First Approach Mode of the Swiss-Model server [99] which includes ProModII model generation and energy minimization with GROMOS96 [100]. The structural analysis was carried out with the Ramanchandran Plot on Swiss-PDB Viewer software [101] version 4.0.1 and Procheck version 3.5.4 on the Structural Analysis and Verification Server (http://nihserver.mbi.ucla.edu/SAVES/). Molecular visualization was performed with PyMOL v. 0.99 (http://www.pymol.org).

### Induction and Inhibition of Secondary Roots

*A. thaliana* (Columbia-0) seeds were sterilized using a 2.5% sodium hypochlorite for 10 min. Seeds were washed 5 times with sterilized water and stored at 4°C in the dark for 48 hours. Seeds were transferred to MS medium supplemented with 0.5 g/L MES and kept at 22°C, 60–70% humidity and 12/12 photoperiod for 7 days. To assess the dose-dependency of the secondary root inhibition we used different concentrations of the synthetic inhibitor SP600125 (0.01; 0.1; 1.0 and 5 µM). The inhibitory effects of SP600125 were also assessed in 7-day seedlings pre-incubated with 5 µM IAA for 24 hours. Germination and initial post-germination morphological development were monitored by optical microscopy.

## Supporting Information

Figure S1 **Transcriptional profile of AtMps1 across several tissues.** Data was obtained from the Arabidopsis thaliana eFP Browser (http://bar.utoronto.ca/efp/cgi-bin/efpWeb.cgi).
(TIF)

Table S1 **Prediction of AtMps1 phosphorylation sites and comparison with hMps1.** Green: conserved in other plant species; red: experimentally characterized hMps1phosphorylation sites; yellow: protein kinase candidate; CT: C-terminal; PlantPhos [61] and DISPHOS [60] (Default predictor); MUSITE [59] (50% specificity).
(TIF)
