# Supplementary figures and images for: Structural and Functional Characterization of the Protein Kinase Mps1 in Arabidopsis thaliana
Source: PLoS One. 2012 Sep 26;7(9):e45707. doi: 10.1371/journal.pone.0045707 (PMC3458904; doi:10.1371/journal.pone.0045707)

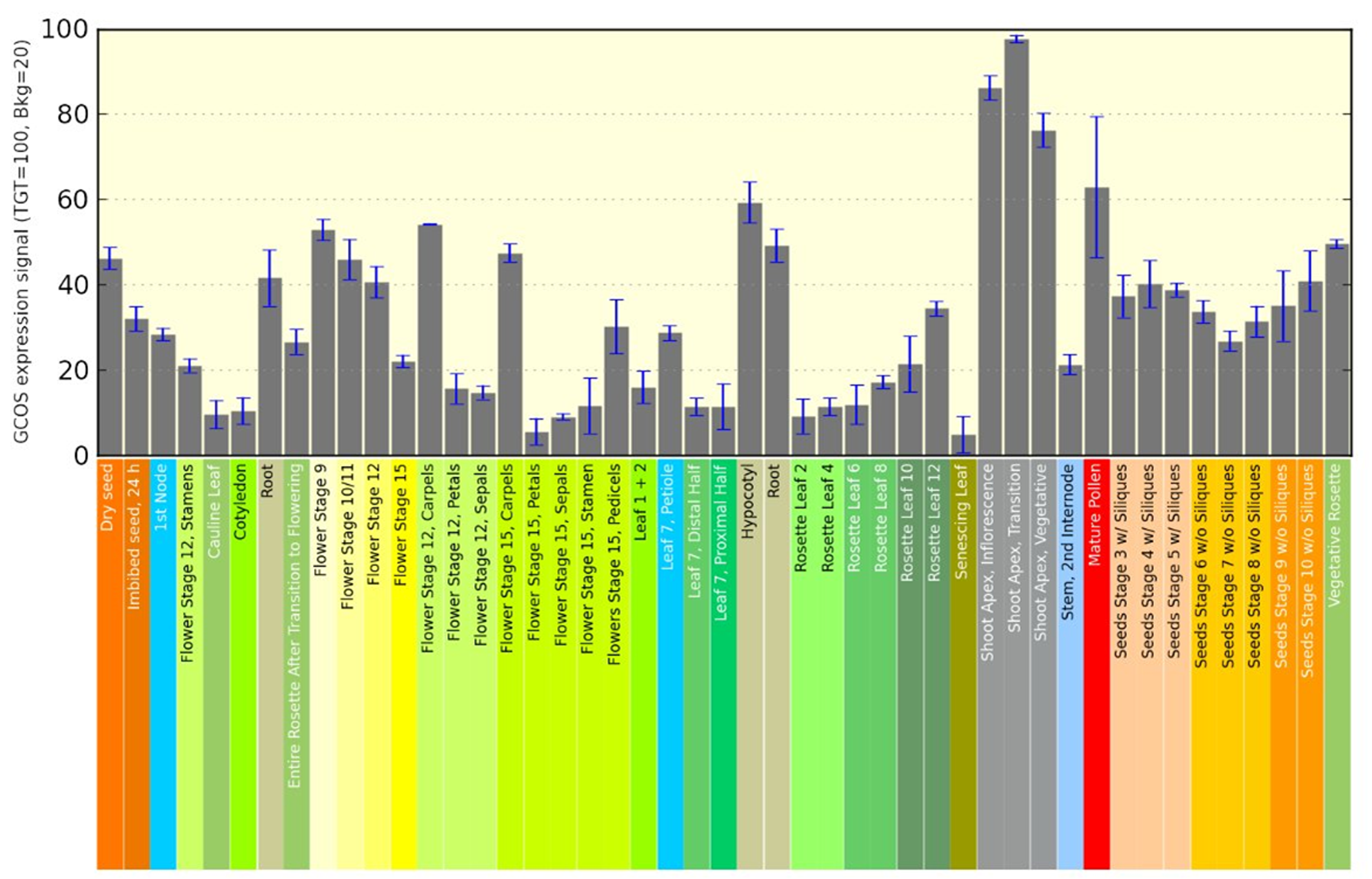

Supplement: Figure S1 — Transcriptional profile of AtMps1 across several tissues. Data was obtained from the Arabidopsis thaliana eFP Browser (http://bar.utoronto.ca/efp/cgi-bin/efpWeb.cgi). (TIF) [file pone.0045707.s001.tif]
